# Long-Term Sex-Dependent Vulnerability to Metabolic challenges in Prenatally Stressed Rats

**DOI:** 10.3389/fnbeh.2017.00113

**Published:** 2017-06-29

**Authors:** Pamela Panetta, Alessandra Berry, Veronica Bellisario, Sara Capoccia, Carla Raggi, Alessia Luoni, Linda Longo, Marco A. Riva, Francesca Cirulli

**Affiliations:** ^1^Center for Behavioral Sciences and Mental Health, Istituto Superiore di SanitàRome, Italy; ^2^Department of Pharmacological and Biomolecular Sciences, University of MilanMilan, Italy

**Keywords:** prenatal stress, high-fat diet, metabolism, sex differences, anxiety, adipokines, *Bdnf*, animal models

## Abstract

Prenatal stress (PNS) might affect the developmental programming of adult chronic diseases such as metabolic and mood disorders. The molecular mechanisms underlying such regulations may rely upon long-term changes in stress-responsive effectors such as Brain-Derived Neurotrophic Factor (BDNF) that can affect neuronal plasticity underlying mood disorders and may also play a role in metabolic regulation. Based upon previous data, we hypothesized that PNS might lead to greater vulnerability to an obesogenic challenge experienced at adulthood. In order to investigate our hypothesis, pregnant Sprague-Dawley female rats underwent a chronic procedure of restraint stress during the last week of gestation. The adult offspring were then challenged with a high fat diet (HFD) over 8 weeks and tested for metabolic and emotional endpoints. Moreover, brain specific changes in *Bdnf* expression levels were also assessed. Overall, HFD resulted in increased caloric intake, insulin resistance, impaired glucose tolerance and higher circulating levels of leptin, while PNS increased the leptin/adiponectin ratio, an index of metabolic risk in adult male subjects. Interestingly, HFD consumption increased anxiety-like behaviors in the Elevated Plus Maze, particularly in males, and this effect was buffered by PNS. Levels of *Bdnf* were finely modulated by PNS and HFD in a region- and sex-dependent fashion: female offspring overall showed greater plasticity, possibly mediated through increased total *Bdnf* mRNA expression both in the hippocampus and in the hypothalamus. In conclusion, while the experience of maternal stress during intrauterine life promotes metabolic dysfunction induced by a HFD at adulthood, the interaction between PNS and HFD is positive in male subjects, and in agreement with the match-mismatch hypothesis, resulting in a reduction of anxious behaviors.

## Introduction

A suboptimal intrauterine environment can program adult chronic diseases predisposing the offspring to develop metabolic and mood disorders later in life (Barker, 1995). Indeed, epidemiological and preclinical studies indicate that excessive stress, also experienced as adverse socio-economic conditions, is a well-established risk factor for mood disorders (Bremner et al., 1997; Heim and Nemeroff, 2001; Cirulli et al., 2003, 2009; Ehlert, 2013; Bock et al., 2014; Boersma et al., 2014; Marosi and Mattson, 2014; Turecki et al., 2014; Berry et al., 2015; Entringer et al., 2015). There is also evidence that traumatic experiences can lead to increased risk for obesity, type 2 diabetes (T2D) and metabolic syndrome at adulthood (Lee et al., 2014; Gavrieli et al., 2015; Lin et al., 2015; Maniam et al., 2015; Yam et al., 2015).

Behavioral and metabolic disorders might share common developmental pathways and impinge upon common mediators, including glucocorticoids (GC)—the main adrenocortical stress hormones—and neurotrophins (most notably brain-derived neurotrophic factor—BDNF), both of which are involved in brain plasticity and metabolic regulation (Tamashiro and Moran, 2010; Lee et al., 2014; Mansur et al., 2015). In particular, BDNF is expressed in hypothalamic areas involved in homeostatic control of feeding and behavior and is released upon stressful challenges, playing a critical role in the integration and optimization of behavioral and metabolic responses (Marosi and Mattson, 2014). There are numerous data from animal models indicating that prenatal stress (PNS) alters later life metabolic and behavioral outcomes in the offspring and that these changes are primarily linked to maternal hypothalamic-pituitary-adrenal (HPA) axis perturbation (Darnaudéry and Maccari, 2008). In particular, a suboptimal environment during pregnancy elevates maternal circulating GC that cross the placental barrier, reducing fetal development predisposing the offspring to increased risk of health deficits at adulthood (Maccari et al., 1995; Bellisario et al., 2015; Entringer et al., 2015). Fewer data, however, are available on the role of BDNF in the long-term effects of PNS (Cirulli, 2017). In rodents, PNS has been shown to induce epigenetic modifications in the BDNF gene, and to reduce overall its expression. However, data may differ depending upon sex, developmental stage investigated and whether total *Bdnf* gene expression or the levels of individual exons are measured (Boersma et al., 2014; Luoni et al., 2014, 2016; Cirulli, 2017). Clinical and preclinical studies indicate that prenatal maternal stress that occurs during the sexual differentiation of the fetal brain has sex-dependent effects on brain development within highly sexually dimorphic regions that regulate mood, stress and metabolic function (Goldstein et al., 2016). Sex-dependent effects have been demonstrated in the response to stress, particularly in the case of prenatal stress as males with a history of *in utero* stress are more prone to develop anxiety-like behavior and cognitive alterations (Zuena et al., 2008). Accordingly, a differential sex-dependent response has been observed demonstrating that PNS female and male rats are programmed to respond differently to an imposed restraint stress at adulthood (Bowman et al., 2004). Moreover, brain structures, such as the hippocampus, that play a role in many aspects of metabolic and mood disorders, show a sex-dependent development and are differentially affected by PNS in male and female offspring predisposing toward a disturbed hippocampal neuroplasticity (Darnaudéry and Maccari, 2008; Cirulli, 2017).

Despite the evidence presented above, few efforts have been made to understand the link between behavioral disturbances and long-term consequences of prenatal stress on metabolic and behavioral indices, taking into account both female and male responsiveness, in rodents. Indeed, although sex-dependent effects are present in the literature, these appear at times contradictory. In this study, pregnant Sprague-Dawley female rats underwent a chronic procedure of restraint stress during the last week of gestation previously validated as inducing neuroendocrine, metabolic and behavioral alterations in the offspring (Maccari et al., 1995; Maccari and Morley-Fletcher, 2007; Luoni et al., 2014). Among the mechanisms advocated to explain the link between the environment experienced in utero and disease susceptibility later in life (developmental programming of health and diseases) feto-placental exposure to maternal stress and to excessive amount of GC hormones may play a key role (Seckl, 1998). Indeed, expression levels of the placental enzyme 11ß-hydroxysteroid dehydrogenase type 2 (*11*β*-HSD2*), that allows for controlled fetal exposure to maternal GC, may be decreased upon prenatal stress, severely affecting fetal development. Thus, levels of the placental *11*β*-HSD2* were measured in stressed and control dams and fetal and pups body weights were analyzed. At adulthood, after feeding the offspring high fat diet for 4 weeks, we focused on plasma changes in leptin and adiponectin and hypothalamic expression of their receptors *LepR, AdipoR1*, and *AdipoR2*. Moreover, we evaluated the gene expression of total and long 3′UTR *Bdnf* mRNA in both ventral hippocampus and hypothalamus as this neurotrophic factor is involved in both energy metabolism and neural plasticity underlying anxiety-like behavior (Cirulli and Alleva, 2009; Tamashiro and Moran, 2010; Paternain et al., 2013; Mou et al., 2015).

## Materials and methods

### Animals and experimental design

Twenty-six adult nulliparous female (230–260 g) and 13 male Sprague-Dawley rats (400 g) were purchased from a commercial breeder (Charles River, Calco, Italy). Upon arrival, animals were pair-housed with same sex conspecifics (cage: 37 × 21 × 19 cm) in an air-conditioned room (temperature 21 ± 1°C, relative humidity 60 ± 10%) under a reversed 12/12 h light/dark cycle (lights off from 07:00 a.m. to 07:00 p.m.). Pellet food (Altromin-R, Rieper, Italy) and tap water were continuously available. Following 7 days of habituation, two females and one male were mated for 24 h. Pregnancy was checked monitoring changes in body weight weekly. Pregnant females were randomly assigned to the control (Ctrl) or prenatal stress (PNS) groups. Ctrl females were left undisturbed throughout gestation, whereas PNS females underwent a repeated stress procedure during the last week of gestation (see Section Dams' stress procedure). On gestational day 20 (GD-20), 4 females from each group were sacrificed to assess placental weight and the expression of the *11*β*-HSD2* enzyme, as well as fetal weight. For the remaining females, the date of parturition was considered as post-natal day 0 (PND-0). On PND-1 all pups were weighted and litters culled to an average of 5 male and 5 female pups. On PND-7 and PND-20 female and male pups from 5 Ctrl and 5 PNS litters were weighted and subsequently used for another experiment to avoid confounding effects due to handling. All the remaining offspring were left undisturbed until PND-21 when they were weaned and housed in groups of 2 or 3 same-sex littermates until 2 months of age. At this age, *n* = 20 Ctrl and *n* = 21 PNS males and *n* = 20 Ctrl and *n* = 19 PNS females were further split and fed either high-fat diet (HFD) or control diet (CD) for 8 weeks. Overall, 8 experimental groups were used and composed as follows: *n* = 10 Ctrl-CD, *n* = 10 Ctrl-HFD, *n* = 10 PNS-CD and *n* = 11 PNS-HFD for the male offspring; *n* = 10 Ctrl-CD, *n* = 10 Ctrl-HFD, *n* = 10 PNS-HFD and *n* = 9 PNS-CD for the female offspring. In order to avoid litter effects, siblings were equally distributed in the experimental groups. After 4 weeks on the respective diets, all animals underwent a Glucose Tolerance Test (GTT) and, 5 days later, an Insulin Sensitivity Test (IST). Ten days following metabolic testing an EPM was also performed in order to assess anxiety-like behavior. All experimental procedures were conducted in conformity with the European Directive 2010/63/EU and the Italian legislation on animal experimentation, D.Lgs. 26/2014.

### Dams' stress procedure

Pregnant females (GD-14) were restrained in a transparent Plexiglas cylinder (7.5 × 19 cm) under a bright light (6.500 lux) for 45 min three times daily until the expected day of parturition (GD-21). This time window is a critical period during which the fetal neuroendocrine and metabolic systems rapidly differentiate. Stress sessions were conducted during the dark phase (9:00 a.m.–5:00 p.m.) at different periods during the day in order to prevent habituation to the repeated procedure (Maccari et al., 1995).

### High-fat diet administration

At 2 months of age, Ctrl and PNS offspring were fed *ad libitum* either with HFD (energy: 5.24 kcal/g; composition: fat 60%, carbohydrate 20% and protein 20%) or CD (energy: 3.3 kcal/g; composition: fat 17%, carbohydrate 60% and protein 23%). The HFD diet (D12492) was purchased from Research Diets, Inc., New Brunswick, NJ, USA while the control diet was purchased from Altromin-R, Rieper, Italy. Body weight and food intake were monitored every week for the first four weeks, until metabolic and behavioral testing started. The diet was continued for a total of 8 weeks. Based on food consumption, the caloric intake was calculated as the ratio between the food consumed in 24-h (g) for each cage and the energy content (Kcal/g) of each diet divided by the number of animals per cage.

### Metabolic measurements

#### Glucose tolerance test (GTT)

At PND-90, after 4 weeks on their respective diets, female and male subjects underwent a GTT. Briefly, after an overnight fasting period (6:00 p.m.–9:00 a.m.) basal blood glucose concentration (levels of glycaemia) was measured (time point 0) in peripheral blood by tail nick using a commercial glucometer (StatStrip Glucose Xpress Meter, Nova Medical, A. Menarini Diagnostics, Italy). Immediately after, animals were intra-peritoneally loaded with 2 g/kg body weight D-glucose (10% D glucose solution; Sigma, St. Louis, MO, USA) and glycaemia was measured at 30, 60, 120, and 180 min from the injection.

#### Insulin sensitivity test (IST)

Five days after the GTT, all groups underwent an IST following a 5-h period of starvation (9:00 a.m.–2:00 p.m.). Animals were intra-peritoneally loaded with a 0.75 U/kg solution of human recombinant insulin (Humulin, Eli-Lilly, 100 U/mL) and glycaemia was measured at time point 0 (baseline immediately before the injection) and 30, 60, 120, and 180 min following the IP injection using a commercial glucometer (StatStrip Glucose Xpress Meter, Nova Medical, A. Menarini Diagnostics, Italy).

### Behavioral characterization

Ten days after being tested for metabolic parameters female and male offspring were tested for anxiety-like behavior in an EPM. The apparatus was made of Plexiglas and consisted of two opposite open arms and two arms closed by transparent walls (50 × 10 × 40 cm). Each rat was placed in the central area of the maze and video-recorded for 5 min. Behavioral parameters observed were: frequency of *entries* and *time spent into the open and closed arms*; latency to enter and percentage of entries into the open arms [(open/total) × 100]; frequency and duration of *immobility* and *head dipping* (HEAD, i.e., exploratory movement of head and shoulders over the edge of the maze), *Stretched-attend-posture* (SAP, forward elongation of head and shoulders followed by retraction to original position). Behavioral analysis was carried out by an observer, blind to the experimental condition, using a commercial software (“The Observer 3.0,” Noldus, The Netherlands). At the end of each test session, the apparatus was cleaned using a cotton pad wetted with a 50% solution of ethanol and water. The test was conducted in a quiet room between 9:30 a.m. and 1:30 p.m. (i.e., during rodents' active-period). Illumination was provided by means of two tall floor lamps with translucent shades placed at opposite corners of the room providing an equal light intensity between the open and closed arms. The total luminosity, as measured at the end of each open and closed arm, was 400 lux on average.

### Tissue collection

On GD-20, 4 pregnant females/group were sacrificed and fetuses and placentas immediately removed and weighed. At 4 months of age female and male offspring were weighted and sacrificed by decapitation. Trunk blood was collected into heparinised microcentrifuge tubes, prepared by low-speed centrifugation at 2,500 rpm for 15 min at 4°C to collect plasma for adipokines and triglycerides quantification. The brain was removed and the ventral hippocampus and hypothalamus were dissected out. All the dissected tissues were immediately frozen at −80°C.

### Biochemical analysis

#### Determination of plasma adipokines and triglycerides

The levels of plasma adipokines (leptin and adiponectin) were assessed using Rat Leptin Elisa kit (Abcam®–ab100773, Cambridge, MA) and Rat Adiponectin Elisa kit (Abcam®–ab108784, Cambridge, MA) respectively. Measurements were conducted according to manufacturer's instructions. Briefly, standards, controls, and samples were placed into the wells and incubated 2 h at room temperature. After washing 5 times, the enzyme-linked polyclonal antibody specific for rats adipokines of interest was added to the wells and then, after washing, the substrate solution was added. The enzyme reaction was read at 450 nm (correction wavelength set at 570 nm). The samples values were read off the standard value. Values below the standard curve were excluded from the final analysis. Plasma triglyceride levels were determined by MultiCarein™ triglyceride test strips and by a specific analyzer (Biochemical Systems International, Arezzo, Italy).

#### RNA preparation for qRT-PCR and analysis of mRNA in the placenta, ventral hippocampus, and hypothalamus

Total RNA was isolated using PureZol RNA isolation reagent (Bio–Rad Laboratories, Italy), treated with DNase to avoid DNA contamination and quantified by spectrophotometric analysis. RNA was analyzed by TaqMan qRT-PCR instrument (CFX384 real time system, Bio–Rad Laboratories) using the iScriptTM one-step RT-PCR kit for probes (Bio–Rad Laboratories). Samples were run in triplicate as multiplexed reactions with a normalizing internal control (*36B4* or ß*-Actin*). Relative target gene expression was calculated according to the 2 (−ΔΔC(T)) method. Probe and primer sequences of *Bdnf* long 3′-UTR (Assay id: Rn02531967_s1), *LepR* (Assay id: Rn01433205_m1), *AdipoR1* (Assay id: Rn01483784_m1), and *AdipoR2* (Assay id: Rn01463173_m1) were purchased from Life Technologies (Monza, Italy) and are available on request, while *total Bdnf* (Fwd: AAGTCTGCATTACATTCCTCGA; Rev:GTTTTCTGAAAGAGGGACAGTTTAT; Probe: TGTGGTTTGTTGCCGTTGCCAAG) and *11*β*-HSD2* (Fwd:GAGGATATCAGCCGTGTTCTG; Rev:TTCACCTCCATACATTCGCG; Probe:AGCATTGTTAACCAGACCCCACAGG) gene expression assays were purchased from Eurofins MWG-Operon (Germany).

### Statistical analysis

Data were evaluated by a two-way ANOVA with Diet (HFD vs. CD) and Prenatal condition (PNS vs Ctrl) as between-subject factors, and Time as within-subject repeated-measures factors, when appropriate (GTT, IST, BW, and EPM test). In the EPM, Zones have been treated as within subject factor only when analyzing % time spent in the open arms to properly compare the time spent in the open vs. the closed arms of the maze. For all other behaviors zones have been treated as dependent variables. *Post hoc* comparisons between groups were performed using the Tukey's test. When main effects of sex were found, separate analyses for females and males were performed. A level of probability set at *p* < 0.05 was used as statistically significant. Data are presented graphically as means + SEM. A linear regression model was used to assess the relationship between levels of circulating triglycerides and the L/A ratio, a marker of metabolic risk.

## Results

### Short-term effects of PNS on the placental barrier *11β-HSD2* on pup's body weight

**Figure 1:**
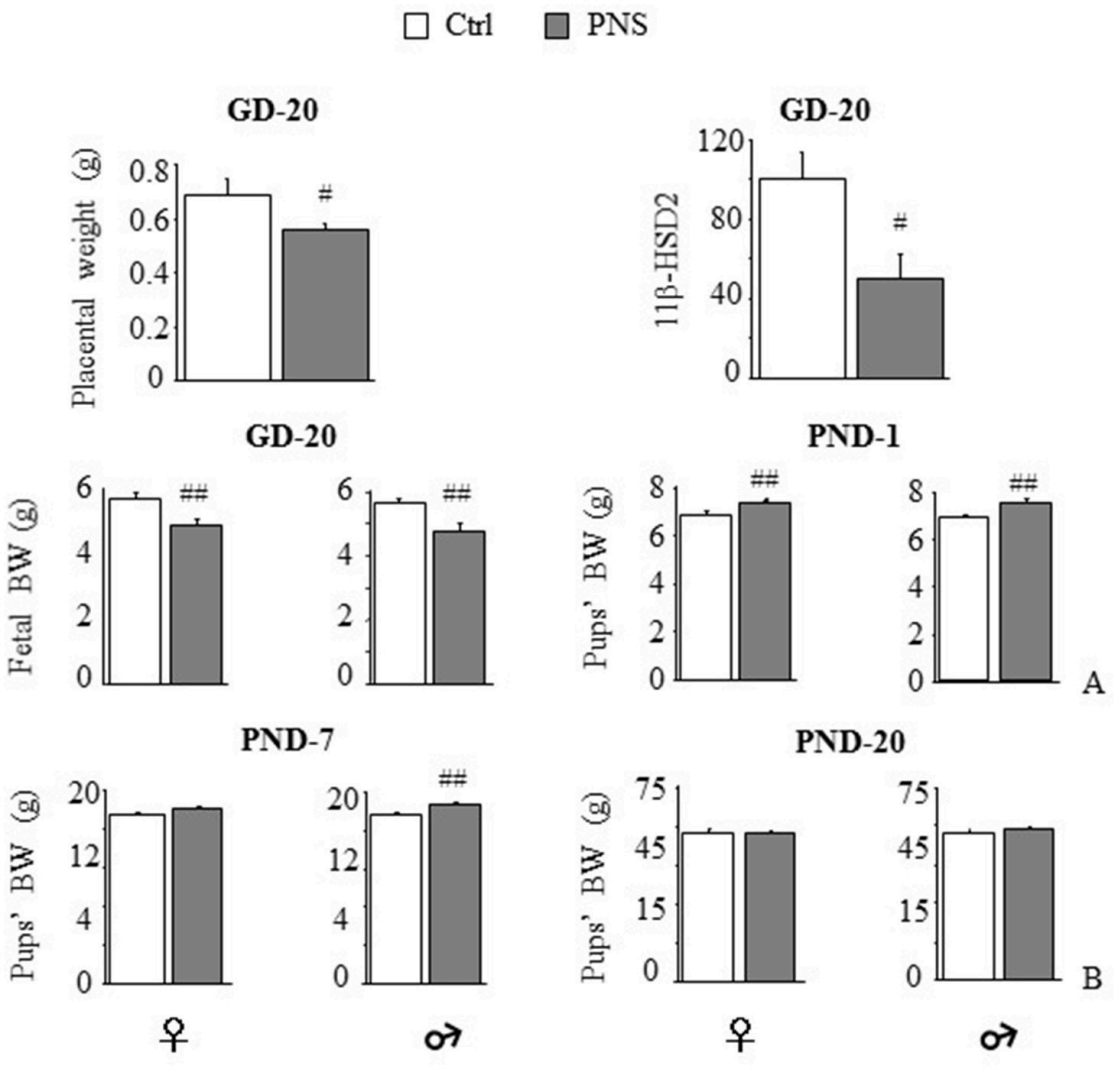
Short-term effects of prenatal stress (PNS) on the placenta and on body weight. PNS reduced placental weight and mRNA expression of the placental barrier enzyme *11*β*-HSD2*. **(A)** PNS reduced fetal body weight (BW) on gestational day (GD) 20; on postnatal day (PND) 1 PNS increased BW in both female and male offspring. **(B)** This effect is maintained on PND-7 only in males while differences in BW disappeared on PND-20. Data are shown as mean + SEM; #*p* < 0.05, ##*p* < 0.01, main effect of Prenatal Condition.

Expression of the placental barrier enzyme *11*β*-HSD2* was reduced in stressed dams [*F*_(1, 11)_ = 7.623, *p* = 0.0185] together with placental weight [*F*_(1, 13)_ = 5.449, *p* = 0.0363] (Figure 1A). No differences were observed in pregnancy length (data not shown). Maternal stress reduced body weight on GD-20 in both female and male fetuses [*F*_(1, 45)_ = 9.846, 8.972; *p* = 0.003, 0.0044]. By contrast, on PND-1, both females and males from prenatally stressed dams were heavier than Ctrl [*F*_(1, 96)_ = 8.207, *p* = 0.0051; *F*_(1, 94)_ = 17.746, *p* < 0.0001 respectively for females and males]. This effect was maintained 7 days after birth in male offspring [*F*_(1, 53)_ = 10.637, *p* = 0.0019], but not in females [*F*_(1, 53)_ = 3.147, *p* = 0.0818] and recovered in both sexes by PND-20 [*F*_(1, 46)_ = 0.001, *p* = 0.9741; *F*_(1, 45)_ = 0.626, *p* = 0.4328; Figure 1B].

### Metabolic data

#### Adult male and female offspring on HFD: body weight and caloric intake

On PND-60, half of the offspring from both Ctrl and PNS groups were fed a HFD. Regardless of PNS, 4 weeks on the diet produced a significant increase of body weight in both sexes compared to CD [main effect of Diet, *F*_(1, 35)_ = 6.694, *p* = 0.0140; *F*_(1, 37)_ = 22.164, *p* < 0.0001, respectively for females and males, and interaction Time × Diet: *F*_(4, 140)_ = 9.854, *p* < 0.0001; *F*_(4, 148)_ = 40.349, *p* < 0.0001, respectively for females and males], subjects showing increased body weight already after 1 week on diet (*p* < 0.01). No interaction was observed between the Prenatal condition and Diet over the 4 weeks [Time × Prenatal Condition × Diet: *F*_(4, 140)_ = 1.08, *p* = 0.3688; *F*_(4, 148)_ = 2.046, *p* = 0.0909 respectively for females and males]. Interestingly, HFD males, but not females, showed a significant increase in caloric intake [*F*_(1, 13)_ = 7.352, *p* = 0.0178; *F*_(1, 12)_ = 0.018, *p* = 0.8942 respectively for males and females] (See Supplementary Figure 1).

#### Glucose tolerance test (GTT) and insulin sensitivity test (IST)

**Figure 2:**
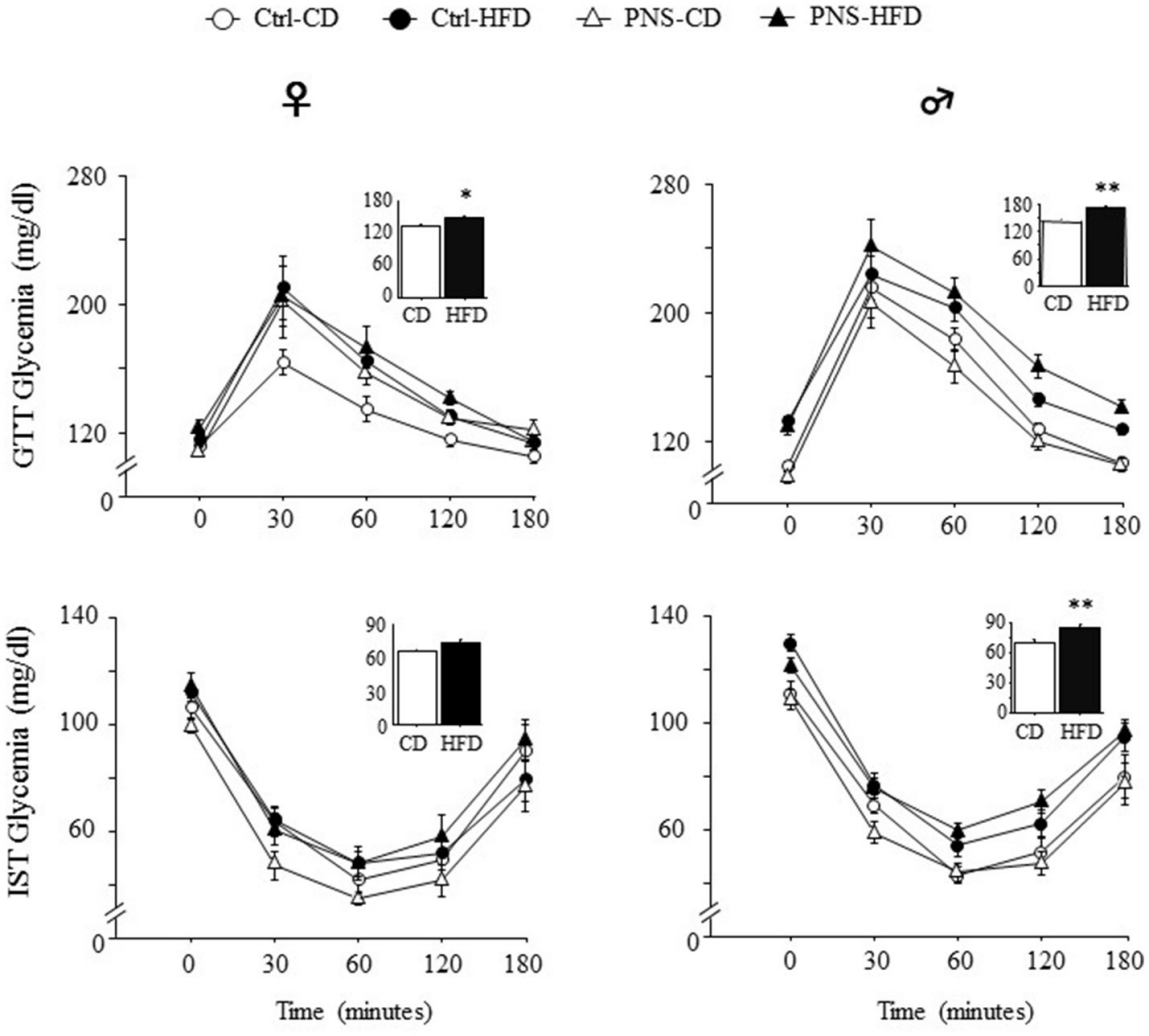
Time course of changes in glycaemia as a result of Glucose tolerance test (GTT) and Insulin sensitivity test (IST) indicate decreased glucose tolerance and insulin sensitivity in subjects fed HFD, with greater effects in males. Data are shown as mean + SEM. ^*^p < 0.05, ^**^p < 0.01, main effect of Diet.

After 4 weeks on the respective diets, PND-90 rats were challenged with a GTT to test glucose clearance. The effect of the bolus injection increased glycaemia in a time-dependent fashion in both sexes [main effect of Time: *F*_(4, 140)_ = 72.980, *p* < 0.0001; *F*_(4, 148)_ = 138.237, *p* < 0.0001, respectively for females and males]. In addition, only HFD fed subjects were characterized by an overt glucose intolerance profile [main effect of Diet: *F*_(1, 35)_ = 5.177, *p* = 0.0291; *F*_(1, 37)_ = 24.690, *p* < 0.0001; respectively for females and males, see Figure 2 upper panel]. No effects of Prenatal Condition alone [*F*_(1, 35)_ = 2.974, *p* = 0.0934; *F*_(1, 37)_ = 0.093, *p* = 0.7620, respectively for females and males] nor in interaction with Diet [*F*_(1, 35)_ = 1.051, *p* = 0.3123; *F*_(1, 37)_ = 2.917, *p* = 0.0960, respectively for females and males] were found to be significant.

Five days after the GTT test, the adult offspring were challenged with an IST to evaluate the response to an insulin injection after 6 h of fasting. Overall, insulin injection decreased glycaemia in a time-dependent fashion in both sexes [main effect of Time *F*_(4, 120)_ = 111.747, *p* < 0.0001; *F*_(4, 164)_ = 173.173, *p* < 0.0001 respectively for females and males]. In addition, only HFD fed males were characterized by an overt glucose intolerance profile [main effect of Diet: *F*_(1, 41)_ = 28.63, *p* < 0.0001] while this was not observed in females [main effect of Diet: *F*_(1, 30)_ = 1.911, *p* = 0.1771, see Figure 2, lower panel]. No effects of Prenatal Condition alone [*F*_(1, 30)_ = 0.476, *p* = 0.4953; *F*_(1, 41)_ = 0.0003, *p* = 0.9862, respectively for female and males] or in combination with Diet [*F*_(1, 30)_ = 3.607, *p* = 0.0672, *F*_(1, 41)_ = 0.722, *p* = 0.4004, respectively for female and males] were observed in both sexes.

#### Plasmatic leptin and adiponectin levels

**Figure 3:**
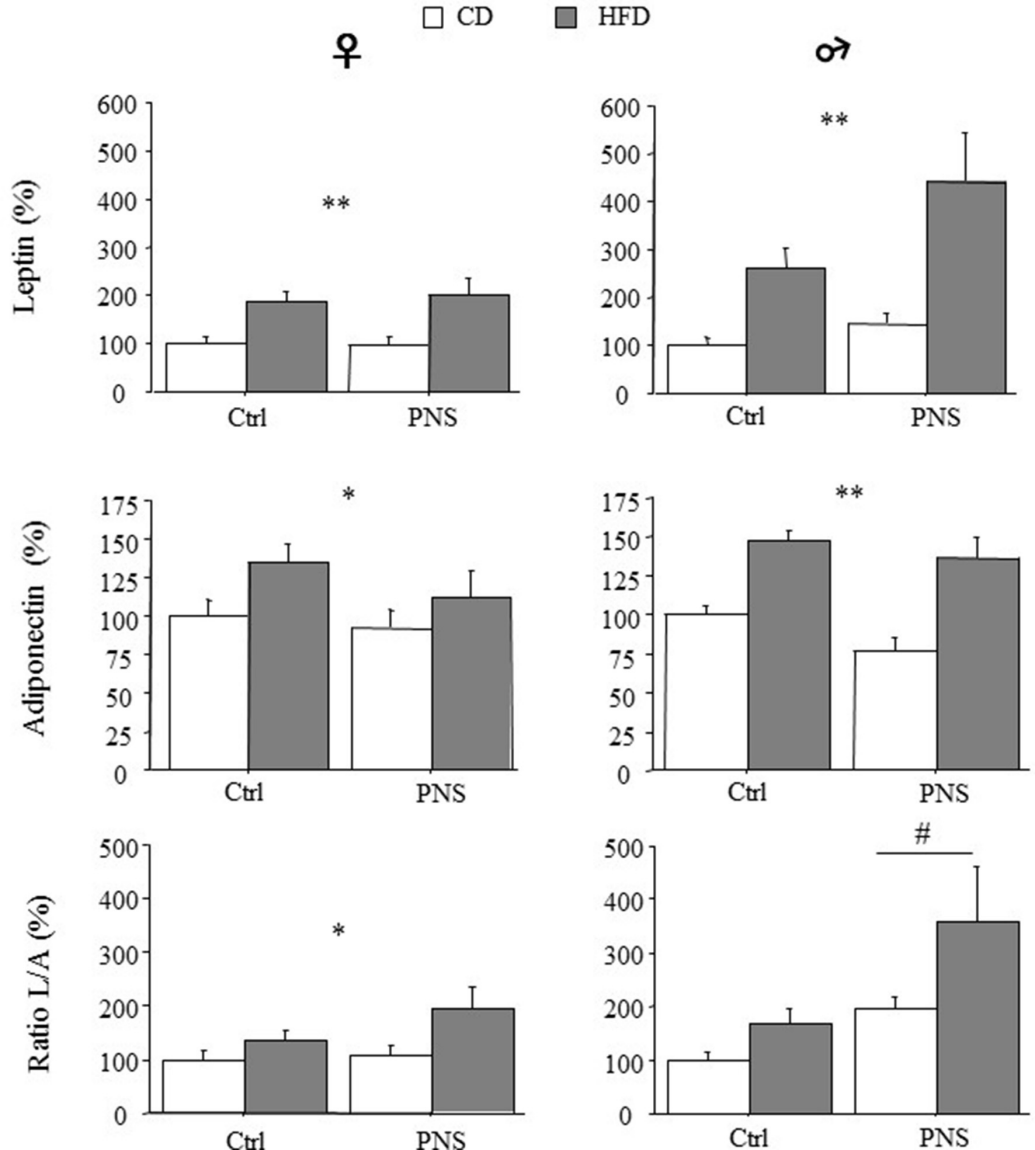
High-fat diet increased relative (% of control) circulating leptin and adiponectin levels in both female and male subjects (HFD vs. CD). High fat diet feeding increased the leptin/adiponectin (L/A) ratio - an index of metabolic risk - in females (CD vs. HFD) but in males a greater increase was observed as a result of Prenatal Condition (PNS vs. Ctrl). Data are presented as means + SEM. Data are shown: ^*^p < 0.05; ^**^p < 0.01, main effect of HFD; #*p* < 0.05, main effect of Prenatal Condition.

A significant increase in circulating adipokines was observed in both females and males as a result of HFD [main effect of Diet: *F*_(1, 35)_ = 15.620, *p* = 0.0004; *F*_(1, 35)_ = 13.054, *p* = 0.0009 for leptin in females and males respectively; *F*_(1, 35)_ = 4.563, *p* = 0.0397; *F*_(1, 35)_ = 28.914; *p* < 0.0001 for adiponectin in females and males respectively, Figure 3]. In male subjects, Prenatal Condition did not affect plasma leptin [*F*_(1, 35)_ = 3.212; *p* = 0.0818] or adiponectin levels [*F*_(1, 35)_ = 3.196; *p* = 0.0825]. However, when the leptin/adiponectin (L/A) ratio was taken into account as marker of metabolic risk, a main effect of Prenatal Condition showed that there was a significant increase in this parameter only in male subjects [*F*_(1, 35)_ = 5.520, *p* = 0.0246, Figure 3]. As for females, no effect of Prenatal Condition was observed [F_(1, 35)_ = 0.068, *p* = 0.7951 and *F*_(1, 35)_ = 1.358, *p* = 0.2518, respectively for leptin and adiponectin] however, a significant increase in the L/A ratio was found in response to HFD [main effect of Diet: *F*_(1, 35)_ = 5.897, *p* = 0.0204, Figure 3]. No interaction effects were found.

#### Plasmatic triglycerides

**Figure 4:**
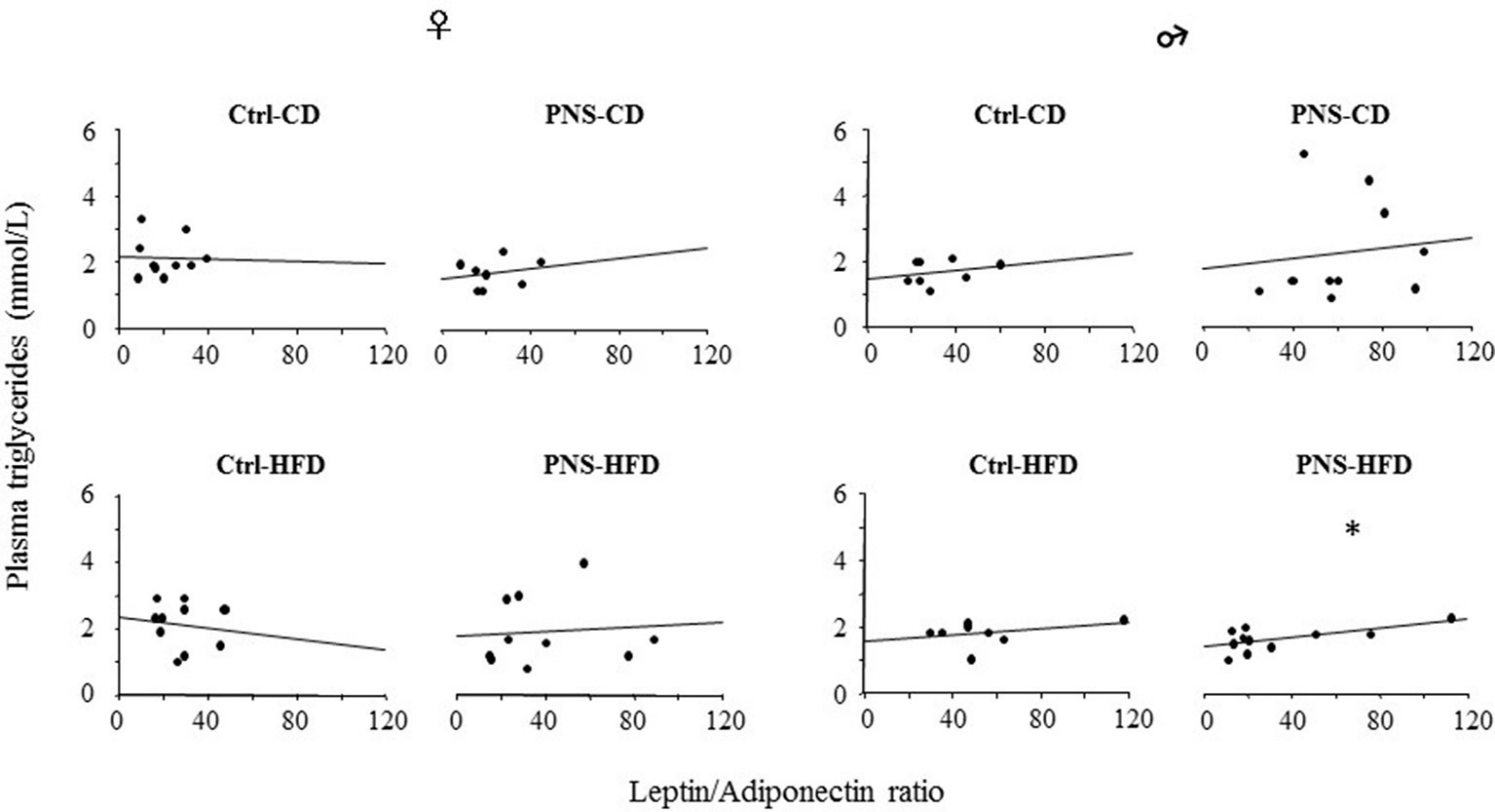
The L/A ratio, a marker of metabolic risk, correlates positively with circulating triglyceride levels only in PNS-HFD males suggesting that a double insult might predispose to a condition of metabolic vulnerability in this sex. The graph represents a linear regression plot. ^*^p < 0.05; *R*^2^ = 0.384.

No differences were observed in the levels of plasma triglycerides in females [*F*_(1, 34)_ = 0.148, 1.584, 0.182; *p* = 0.7028, 0.2168, 0.6725 respectively for Diet, Prenatal Condition and Diet × Prenatal Condition] nor in males [*F*_(1, 35)_ = 1.826, 0.264, 1.721; *p* = 0.1852, 0.6103, 0.1982 respectively for Diet, Prenatal Condition and Diet × Prenatal Condition]. However, the L/A ratio showed a positive correlation with plasma triglycerides in PNS-HFD males [*F*_(1, 10)_ = 5.614, *p* = 0.0419, *R*^2^ = 0.384] but not in the other groups of males [Ctrl-CD: *F*_(1, 7)_ = 0.401, *p* = 0.5502; *R*^2^ = 0.063; PNS-CD: *F*_(1, 9)_ = 0.113; *p* = 0.7457; *R*^2^ = 0.014; Ctrl-HFD: *F*_(1, 7)_ = 0.809; *p* = 0.4032; *R*^2^ = 0.119] nor in females [Ctrl-CD: *F*_(1, 9)_ = 0.007; *p* = 0.9343; *R*^2^ = 0.001; PNS-CD: *F*_(1, 7)_ = 0.337; *p* = 0.5825; *R*^2^ = 0.053; Ctrl-HFD: *F*_(1, 9)_ = 0.137; *p* = 0.7213; *R*^2^ = 0.017; PNS-HFD: *F*_(1, 9)_ = 0.057; *p* = 0.8174; *R*^2^ = 0.007; Figure 4].

### Behavioral effects

#### Elevated Plus Maze (EPM)

**Figure 5:**
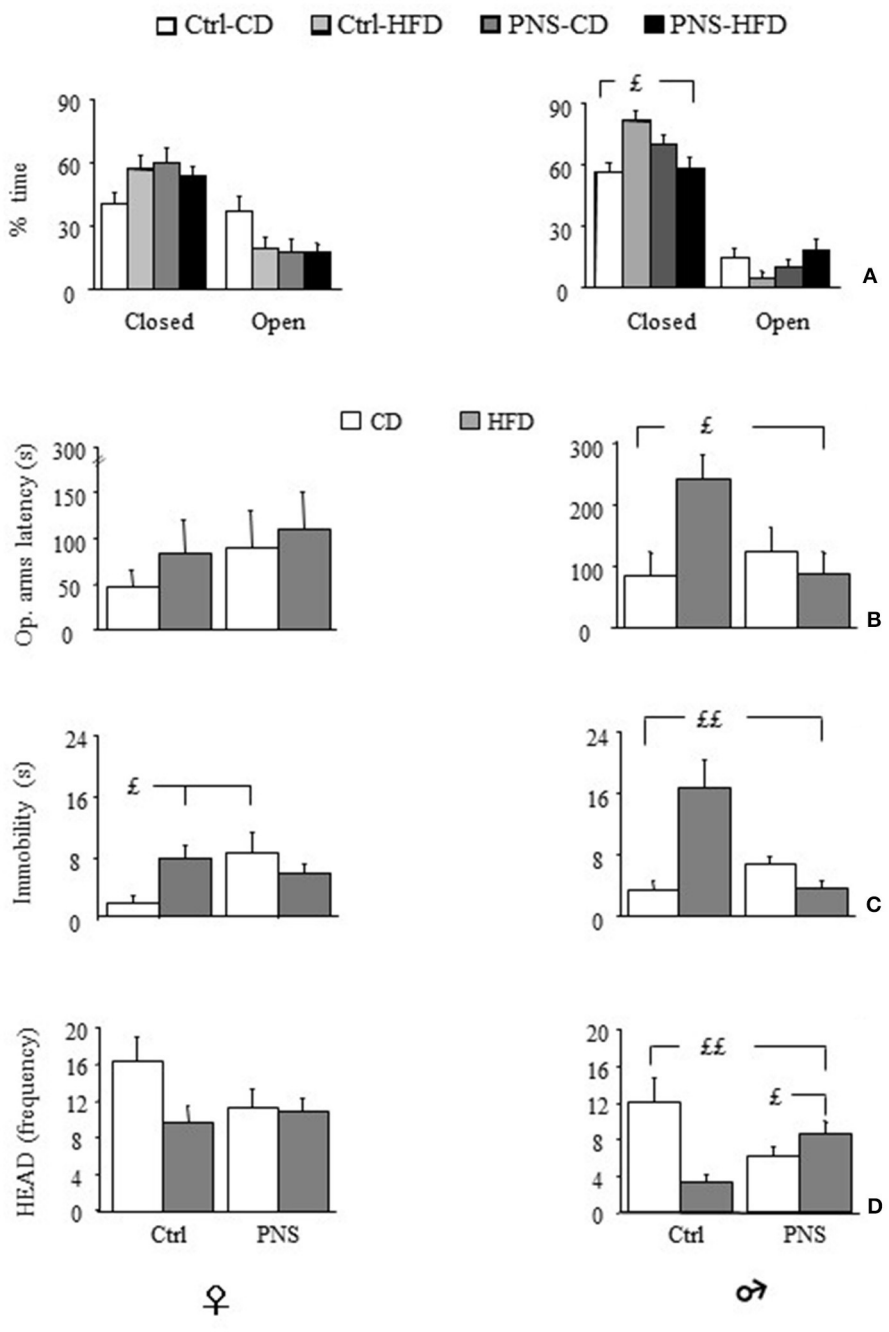
Anxiety-like behavior in the Elevated Plus Maze (EPM). **(A)** Time (%) spent in closed vs. open arms: HFD feeding increases anxiety levels in male subjects however, PNS buffers this effect reducing the time spent in the closed arms of the maze (Ctrl-HFD vs. Ctrl-CD and PNS-HFD); **(B)** Latency to enter the open arms is increased by HFD only in males though PNS is able to prevent this increase (Ctrl-HFD vs. Ctrl-CD and PNS-HFD); **(C)** In male subjects, immobility is specifically increased by HFD though PNS is able to prevent this increase (Ctrl-HFD vs. Ctrl-CD and PNS-HFD); by contrast, in females both HFD and PNS increase the time spent immobile (Ctrl-HFD and PNS-CD vs. Ctrl-CD). **(D)** Head dipping (HEAD), a measure of exploration, is decreased by both HFD and prenatal stress only in males. Exposure to the double hit (PNS-HFD) prevents this decrease (Ctrl-HFD and PNS-HFD vs. Ctrl-CD and PNS-CD vs. Ctrl-CD). Data are presented as means + SEM. £*p* < 0.05; ££*p* < 0.01, interaction between Prenatal Condition and Diet.

Overall, all subjects spent more time in the closed arms of the maze [*F*_(1, 35)_ = 28.504, *p* < 0.0001; *F*_(1, 37)_ = 137.202, *p* < 0.0001 respectively for females and males]. Analysis of total arm entries—an indirect measure of locomotor activity—showed no difference as a result of treatment or diet [males: *F*_(1, 37)_ = 0.111, 1.370, 1.485; *p* = 0.7404, 0.2494, 0.2307 respectively for prenatal condition, diet and prenatal condition × diet interaction; females: *F*_(1, 35)_ = 0.435, 0.009, 0.258; *p* = 0.5141, 0.9236, 0.6146 respectively for Prenatal condition, Diet and Prenatal condition × Diet interaction]. Overall, Ctrl-HFD male subjects showed a greater percent time spent in the protected part of the maze (closed arms) [*F*_(1, 37)_ = 4.598, *p* = 0.0386]. This was not observed in females [*F*_(1, 35)_ = 1.648, *p* = 0.2076]. Interestingly, this effect was prevented in PNS-HFD males (Zone × Prenatal Condition × Diet: *F*_(1, 35)_ = 3.192, *p* = 0.0826; *F*_(1, 37)_ = 8.749, *p* = 0.0054 respectively for females and males; *post hoc* comparisons for males Ctrl-HFD vs. PNS-HFD *p* < 0.05, see Figure 5A). Moreover, Ctrl-HFD male rats took longer to start exploring the anxiogenic portion of the maze [Prenatal Condition × Diet interaction: *F*_(1, 35)_ = 0.063, *p* = 0.8027 and *F*_(1, 37)_ = 6.394, *p* = 0.0158, respectively for females and males; *post hoc* comparisons for males: Ctrl-HFD vs. Ctrl-CD and PNS-HFD, *p* < 0.05; Figure 5B]. A significant increase in immobility was observed both in Ctrl-HFD and PNS-CD females when compared to Ctrl-CD subjects [Prenatal Condition × Diet interaction: *F*_(1, 35)_ = 5.859, *p* = 0.0208; *post hoc* comparisons, *p* < 0.05, Figure 5C). This increased immobility was also observed in Ctrl-HFD male subjects, although prenatal stress prevented this effect [*F*_(1, 37)_ = 17.821, *p* = 0.0002, *post hoc*: *p* < 0.01 Ctrl-HFD vs. Ctrl-CD and PNS-HFD Figure 5C]. As for HEAD, no main effect of Diet [*F*_(1, 35)_ = 3.011, *p* = 0.0915] or Prenatal Condition [*F*_(1, 35)_ = 0.958, *p* = 3.3345] or of their interaction [Prenatal condition × Diet: *F*_(1, 35)_ = 2.314, *p* = 0.1372] was observed in females. By contrast, Ctrl-HFD males were characterized by a decrease in the frequency of this exploratory behavior that was prevented in PNS-HFD subjects [Prenatal Condition × Diet: *F*_(1, 37)_ = 12.657, *p* = 0.001 *post hoc*: Ctrl-HFD vs. Ctrl-CD and PNS-HFD, *p* < 0.01; PNS-HFD vs. PNS-CD, *p* < 0.05 Figure 5D]. As for SAP no effect was observed as a result of Diet, Prenatal condition or as a combination of these factors in females [Latency: *F*_(1, 35)_ = 0.0001575, 0.2533, 0.392; *p* = 0.9901, 0.1205, 0.5354; Frequency: *F*_(1, 35)_ = 0.176, 0.384, 0.587; *p* = 0.6772, 0.5392, 0.4486; Duration: *F*_(1, 35)_ = 0.050, 0.208, 1.192; *p* = 0.8238, 0.6510, 0.2825, respectively for Diet, Prenatal Condition and Diet × Prenatal Condition] nor in male rats [Latency: *F*_(1, 37)_ = 2.775, 3.573, 2.105; *p* = 0.1042, 0.0666, 0.1552; Frequency: *F*_(1, 37)_ = 0.063, 0.724, 2.216; *p* = 0.8038, 0.4004, 0.1451; Duration: *F*_(1, 37)_ = 0.016, 0.016, 3.304; *p* = 0.8992, 0.9012, 0.0772 respectively for Diet, Prenatal Condition and Diet × Prenatal Condition].

### CNS gene expression

Total *Bdnf* mRNA expression and long 3′ UTR *Bdnf* mRNA were measured in the ventral hippocampus and hypothalamus of both females and males 10 days after behavioral testing. The expression of *LepR, AdipoR1*, and *AdipoR2* mRNA has also been measured in the hypothalamus.

#### Gene expression of total *Bdnf* and long 3′ UTR *Bdnf* mRNA in the ventral hippocampus and hypothalamus

**Figure 6:**
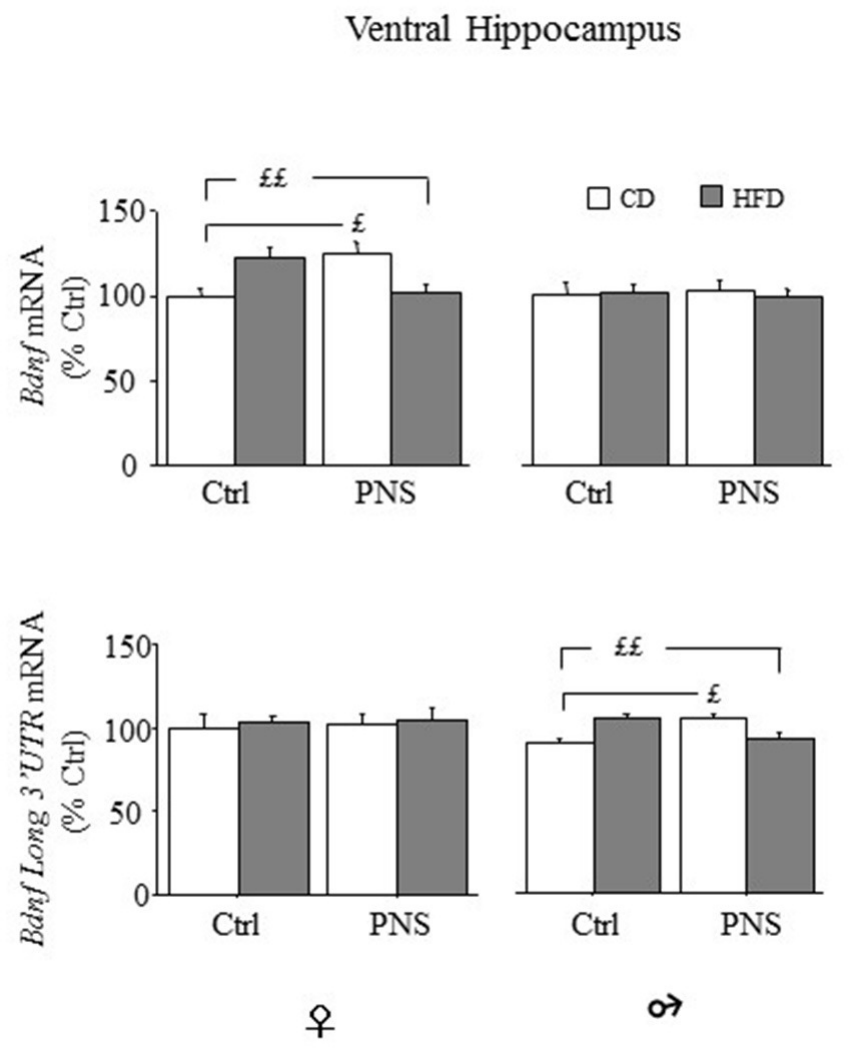
*Bdnf* mRNA expression in the ventral hippocampus of female and male offspring. Relative mRNA levels (% ctrl) of total *Bdnf* (upper panel) and of *Bdnf long 3′UTR* mRNA (lower panel). In females both PNS and HFD increased total *Bdnf* mRNA while exposure to PNS in HFD-fed rats prevented this increase (Ctrl-HFD vs. Ctrl-CD and PNS-HFD; PNS-CD vs. Ctrl-CD). Comparable effects can be observed in male rats for the levels of *Bdnf long 3*′ *UTR* (Ctrl-HFD vs. Ctrl-CD and PNS-HFD; PNS-CD vs. Ctrl-CD). Data are shown as mean + SEM. ££*p* < 0.01 £*p* < 0.05, interaction between Prenatal Condition and Diet.

In the ventral hippocampus, as far as total *Bdnf* mRNA levels, no main effect of Diet or Prenatal Condition was found [main effect of Diet, *F*_(1, 35)_ = 0.014, *p* = 0.9077, and *F*_(1, 36)_ = 0.067, *p* = 0.7965 respectively for females and males; main effect of Prenatal Condition, *F*_(1, 35)_ = 0.130, *p* = 0.7205, and *F*_(1, 36)_ = 0.8927, *p* = 0.9925, respectively for females and males]. However, in female subjects, a significant Diet × Prenatal Condition interaction [*F*_(1, 35)_ = 19.317, *p* < 0.0001] revealed higher *Bdnf* levels in Ctrl-HFD rats when compared to both Ctrl-CD and PNS-HFD (*post hoc*: *p* < 0.01) and in PNS-CD when compared to Ctrl-CD (*p* < 0.05); significant effects of these interactions were not observed in male rats [*F*_(1, 36)_ = 0.142, *p* = 0.7083, see Figure 6, upper panel]. With regard to the expression of the long 3′ UTR *Bdnf* transcript, in female subjects, no effect of Diet [*F*_(1, 34)_ = 0.179, *p* = 0.6751], of Prenatal Condition [*F*_(1, 34)_ = 0.082, *p* = 0.7766] nor an interaction between these factors was observed in the ventral hippocampus [*F*_(1, 34)_ = 0.006, *p* = 0.9365]. In males, no effect of Diet [*F*_(1, 36)_ = 0.023, *p* = 0.8805] and of Prenatal Condition [*F*_(1, 36)_ = 0.124, *p* = 0.7268] were observed. However, a significant Prenatal Condition x Diet interaction [*F*_(1, 36)_ = 6.195, *p* = 0.0176] was observed. In particular *post hoc* comparisons revealed higher *Bdnf* long 3′ UTR mRNA levels in Ctrl-HFD rats when compared to both Ctrl-CD and PNS-HFD (*post hoc*: *p* < 0.01) and in PNS-CD when compared to Ctrl-CD (*p* < 0.05) (see Figure 6, lower panel).

**Figure 7:**
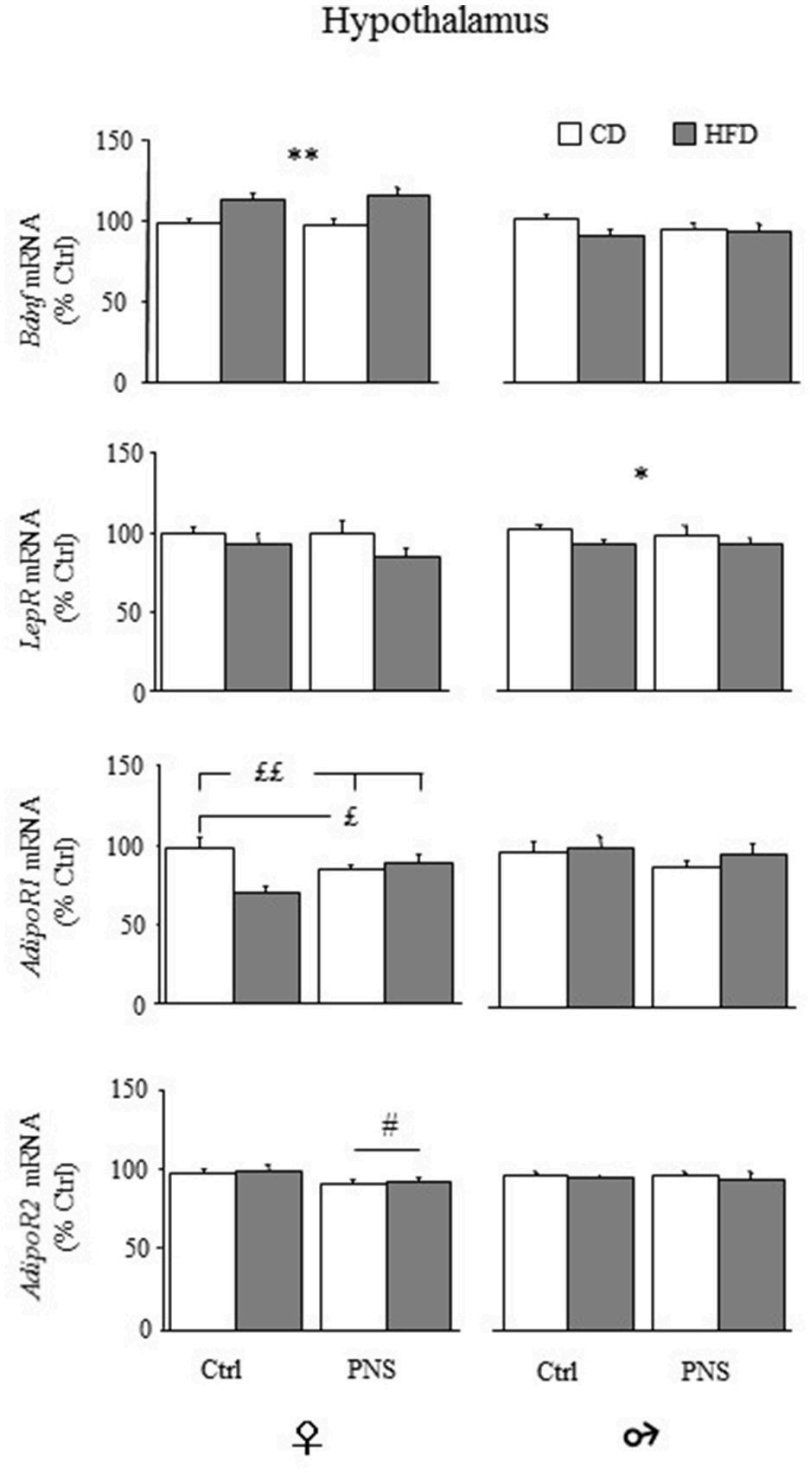
Relative mRNA levels (% ctrl) of hypothalamic *Bdnf*, leptin (*LepR*) and adiponectin receptors (*AdipoR1* and *AdipoR2*). Total *Bdnf* mRNA levels were increased only in females as a result of HFD feeding (HFD vs. CD). *LepR* mRNA levels were reduced only in male rats following HFD (HFD vs. CD). *AdipoR1* mRNA was reduced both in Ctrl-HFD as well as in PNS-CD females while PNS-HFD subjects showed *AdipoR1* levels comparable to those observed in Ctrl-CD rats (Ctrl-HFD vs. Ctrl-CD and PNS-HFD; PNS-CD vs. Ctrl-CD). *AdipoR2* levels were increased only in females as a result of HFD feeding (HFD vs. CD). Data are shown as mean + SEM. ^*^p < 0.05, ^**^p < 0.01 main effect of Diet; £*p* < 0.05, ££*p* < 0.01, interaction between Prenatal Condition and Diet; #<0.05, main effect of Prenatal Condition.

In the hypothalamus, females fed HFD showed increased expression of total *Bdnf* mRNA compared to females fed CD [main effect of Diet, *F*_(1, 34)_ = 18.383, *p* = 0.0001, see Figure 7, upper panel), Prenatal Condition having no effect [*F*_(1, 34)_ = 0.040, *p* = 0.8430]. No changes were observed in males as a result of HFD [main effect of Diet: *F*_(1, 37)_ = 2.390, *p* = 0.1306] or as a result of Prenatal Condition [*F*_(1, 37)_ = 0.240, *p* = 0.6274]. No effects of Prenatal Condition × Diet were observed in either male or female rats. In both sexes there were no differences in the hypothalamic expression of long 3′-UTR *Bdnf* mRNA (data not shown). No significant correlation with behaviors indicative of anxiety in the EPM was found (data not shown).

#### Hypothalamic expression of adipokine's receptors mRNA

Overall, hypothalamic expression levels of *LepR* mRNA were significantly reduced in males fed HFD [main effect of Diet, *F*_(1, 37)_ = 4.185; *p* = 0.048], but not in females [main effect of Diet, *F*_(1, 34)_ = 3.492, *p* = 0.070; Figure 7]. No effects of Prenatal Condition were found in both sexes (main effects of Prenatal stress [*F*_(1, 34)_ = 0.488; *p* = 0.4896 for females and *F*_(1, 37)_ = 0.094; *p* = 0.7607 for males].

With regard to AdipoR1, in female subjects, a main effect of Diet was found, with a significant reduction in the expression of the hypothalamic *AdipoR1* [*F*_(1, 35)_ = 5.777, *p* = 0.0217; Figure 7]. No main effect of Prenatal Condition was found [*F*_(1, 35)_ = 0.190, *p* = 0.6658] while a significant Diet × Prenatal Condition interaction [*F*_(1, 35)_ = 12.368, *p* = 0.0012] showed a specific decrease in the levels of *AdipoR1* in Ctrl-HFD, when compared to both Ctrl-CD and PNS-HFD (*post hoc*: *p* < 0.01), as well as in PNS-CD, when compared to Ctrl-CD (*post hoc: p* < 0.05). In males, no effect of Diet [*F*_(1, 36)_ = 0.735, *p* = 0.3968], of Prenatal Condition [*F*_(1, 36)_ = 1.279, *p* = 0.2656] nor an interaction between these factors was found (Figure 7).

As for *AdipoR2*, females showed a significant reduction in its expression as a result of PNS [main effects of Prenatal Condition: *F*_(1, 35)_ = 5.380, *p* = 0.0263] while HFD exposure showed no effect [*F*_(1, 35)_ = 0.234, *p* = 0.6316]; no interaction effects were observed. In males, no main effect of Diet [*F*_(1, 37)_ = 0.661, *p* = 0.4215] or of Prenatal Condition [*F*_(1, 37)_ = 0.001, *p* = 0.9805] and no interaction effects were found (Figure 7).

## Discussion

In the present study we tested the hypothesis that maternal stress imposed on the fetus during the last week of gestation might perturb the intrauterine environment, programming, in a sex-dependent manner, the offspring toward mal (adaptive) metabolic and behavioral alterations in response to a metabolic stress—represented by HFD—encountered later in life.

Overall, our results suggest that males are more vulnerable than females to the effects of a HFD challenge from a metabolic and behavioral standpoint. In particular, here we show that: (1) immobilization stress during pregnancy led to a reduction in placental weight and *11*β*-HSD2* mRNA expression, fetal growth retardation and to abnormal body weight up to the juvenile period only in males (PND-7) as a possible result of sex-dependent developmental perturbation induced by PNS and that (2) when the L/A metabolic risk marker was analyzed at adulthood, PNS induced a strong vulnerability to HFD in males but not in females; in general, exposure to HFD increased leptin and adiponectin blood levels in both sexes and led to reduced insulin sensitivity and glucose tolerance, with greater effects in the male offspring. In addition, males, but not females, showed increased caloric intake and reduction of the hypothalamic *LepR* induced by the diet, suggesting a central leptin resistance, while females showed central adaptation to the adiponectin signal in response to both PNS and HFD; (3) in males, HFD resulted in increased anxiety in the EPM, which was buffered in those subjects previously exposed to PNS; (4) in females, HFD led to higher total *Bdnf* mRNA expression in the hypothalamus and ventral hippocampus, an indirect indication of greater plasticity in response to metabolic challenges in this sex.

### Short-term perturbations induced by PNS

In rats, alterations of the HPA axis, induced by PNS or by synthetic glucocorticoids administration during late pregnancy, result in offspring with low birth weight, glucose intolerance, insulin resistance and hypertension (Lindsay et al., 1996; Lesage et al., 2004). We found a reduction of placental and fetal weight on GD-20, both of which are macroscopic signs of the developmental perturbations induced by PNS (Lesage et al., 2004). Accordingly, we also observed a reduction in the expression of placental *11*β*-HSD2* that, as already suggested, may increase the trans-placental passage of maternal glucocorticoids reducing fetal growth and birth weight in both humans and rodent in comorbidity with later metabolic disturbance and anxiety-related behaviors (Lindsay et al., 1996). Partially in contrast with these studies, we observed that offspring of stressed dams show increased body weight at birth (PND-1) compared to controls. However, some studies showed an increased birth weight due to PNS (Tamashiro and Moran, 2010) or no differences compared to control (D'mello and Liu, 2006) suggesting that the impact of PNS on birth weight depends upon stress type, duration, timing or experimental model used. We can hypothesize that, in our study, the body weight gain on PND-1 in prenatally stressed offspring compensates the reduced fetal weight observed on GD-20. Interestingly, this abnormality persists during the juvenile period (PND-7) only in males indicating that the sex-dependent programming exerted by an intrauterine stress is already present early during postnatal life and might be linked to differential sensitivity of the placenta to glucocorticoids in males vs. females, both in animal models and in humans (Mueller and Bale, 2006).

### Long-term effects of prenatal stress on metabolic parameters and hypothalamic *Bdnf* mRNA expression

Males appeared more vulnerable to the effects of PNS and HFD compared to females. Only males increased significantly caloric intake following HFD and showed a greater increase in body weight, compared to females. Insulin resistance is associated with hyperinsulinemia and, although we did not measure circulating insulin levels, we found reduced insulin sensitivity and reduced glucose tolerance in HFD-fed subjects, particularly in males. In addition, HFD increased circulating leptin and adiponectin in both females and males, but the L/A ratio, a reliable marker of metabolic risk that has been also related to insulin resistance (Zaletel et al., 2010; Lubkowska et al., 2015), was significantly increased by PNS only in males.

Sex-differences in leptin and adiponectin levels could play a pivotal role in mediating the metabolic alterations observed. Studies in humans and rodents have shown that females have higher basal levels of adiponectin than males, the expression of this adipokine being under the control of sexual hormones (Nishizawa et al., 2002). In our study, the sexual dimorphism in the circulating levels of adipokines is reflected in the analysis of the leptin/adiponectin (L/A) ratio. A growing body of evidence suggests that the L/A ratio may be considered as a sensitive risk marker of metabolic syndrome in patients with overweight and obesity and has been also suggested to be a reliable indicator of insulin resistance (Zaletel et al., 2010; Labruna et al., 2011; Lubkowska et al., 2015). In addition, this ratio has been found to be finely modulated by sex hormones as for example androgens may affect both leptin and adiponectin levels (Lubkowska et al., 2015) and testosterone in particular has been shown to decrease adiponectin levels in rats (Nishizawa et al., 2002). In this regard, it is worth noticing that PNS is able to affect many aspects of sexual differentiation, including the concentrations of reproductive hormones (Ashworth et al., 2016). Thus, although we did not assess directly these parameters, we cannot exclude that PNS might set the ground for vulnerability to metabolic dysfunction by modulating sex hormones. Moreover, the L/A ratio correlated positively with triglyceride blood levels in PNS males fed HFD, but not in females, suggesting a sex-related vulnerability toward metabolic alterations due to suboptimal prenatal conditions and diet (Labruna et al., 2011; Paternain et al., 2013).

Peripheral changes induced by HFD were mirrored by changes in CNS mediators and maintained in a sex-dependent fashion. Leptin acts on the hypothalamus through its receptors (*LepR*) mainly to reduce food intake and body weight (Sohn et al., 2013). In our study, male rats fed HFD, but not females, show reduced expression of the hypothalamic *LepR* suggesting a leptin resistance that may predispose males toward an increased caloric intake and metabolic alterations. On the contrary, females showed less pronounced peripheral changes in adipokines accompanied to changes in hypothalamic *AdipoR1* and *AdipoR2* mRNA. Females fed HFD showed a down-regulation in the hypothalamic expression of *AdipoR1*, potentially attributable to the increase of circulating adiponectin, which was prevented by PNS. It is possible to hypothesize that all these changes represent an adaptive mechanism, together with reduced *AdipoR2* expression, not present in males, which may “preserve” HFD females from the deleterious effects of an obesogenic diet.

Hypothalamic BDNF plays a major role in metabolic regulation. BDNF is an important anorexigenic factor able to reduce appetite and to increase energy expenditure (Vanevski and Xu, 2013). Injections of BDNF in the paraventricular nucleus (PVN) of obese rats fed HFD normalize energy intake, body weight, hyperlipidemia, hyperinsulinemia, and hyperleptinemia indicating that hypothalamic BDNF improves metabolic syndrome symptoms associated with insulin and leptin resistance (Wang et al., 2010). Interestingly, BDNF up-regulation in the hypothalamus depends upon leptin receptor signaling (Komori et al., 2006). In our study, as previously discussed, HFD fed male rats showed more serious metabolic alterations than females, accompanied by reduced *LepR* expression and no changes in total *Bdnf* mRNA expression in the hypothalamus. It may be argued that metabolic alterations displayed by HFD fed male rats may be related, at least in part, to the inability of the peripheral leptin signal to up-regulate hypothalamic *Bdnf* mRNA expression, failing to reduce caloric intake and to limit the deleterious consequences of HFD consumption. By contrast, HFD fed female rats showed no changes in food intake and better resilience to the effects of HFD compared to males, a result which may be partially attributable to the up-regulation of total *Bdnf* mRNA in the hypothalamus. Accordingly, a study by Liu and colleagues already suggested a sex-distinct regulation of hypothalamic *Bdnf* expression induced by diet and energy status (Liu et al., 2014). Overall, the increase in hypothalamic total *Bdnf* mRNA exclusively in females suggests that a greater plasticity in the female sex might be mediated by selective changes in this neurotrophin.

### Long-term effects of prenatal stress on anxiety and hippocampal *Bdnf* mRNA expression

Although EPM was the only anxiety test performed and, more in general, the only behavioral test carried out, our observations show strong effects of HFD feeding on males' emotional behavior. In fact, male rats were characterized by increased time spent in the closed arms, immobility duration and latency to enter in the open arms; HFD fed female rats only showed an increase in immobility. Differently from metabolic measures, PNS reduced frequency of behavioral indices of anxiety in males only, as already reported (Darnaudéry and Maccari, 2008). Indeed, it has been previously shown that the effects of PNS on anxiety are less marked in females (Zagron and Weinstock, 2006) and possibly even contrary to those observed in males, as suggested by the decreased anxiety like behavior recently observed in PNS females. The sex effect on behavior seems to be related to a reduction in hippocampal plasticity in PNS males and to an increase in PNS females (Darnaudéry and Maccari, 2008).

These data are interesting if contrasted with changes in *Bdnf* mRNA expression as measured in the hippocampal region. Indeed, as a possible central mechanism mediating the anxiety response to PNS and HFD exposure, we focused on the expression of *Bdnf* mRNA in the ventral hippocampus, a region primarily involved in the regulation of anxiety. Accordingly, in this study we show that female, but not male rats, fed HFD or exposed to PNS displayed increased levels of total *Bdnf* in the ventral hippocampus, an effect prevented by PNS exposure. The same pattern was found only in males when long 3′ UTR *Bdnf* was assayed.

Compared to previous data, here we analyzed changes in *Bdnf* transcripts specific to ventral rather than to the whole hippocampus, and in older subjects, so data appear at variance with previous reports (Luoni et al., 2014), although in a previous paper a tendency for PNS females to show increased levels of total *Bdnf* mRNA was described (Luoni et al., 2014).

Considering that long 3′ UTR *Bdnf* mRNA is thought to contribute to rapid activity-dependent translation of the neurotrophin and that total *Bdnf* mRNA levels were unchanged in PNS male rats, we could speculate of a greater plasticity in females. In line with these considerations, recent studies have indeed indicated an association between circulating adiponectin levels and anxiety-like behavior and depression, both in humans and rodents (Nicolas et al., 2015), an effect potentially mediated by hippocampal mechanisms involving changes in *Bdnf* expression (Yau et al., 2014). It could be argued that, in PNS females, the increase of adiponectin induced by HFD could limit the anxiogenic effects of HFD given alone through changes in hippocampal and hypothalamic *Bdnf* expression. Further studies need to be undertaken to clarify the link among PNS-HFD-emotional behavior and sex-dependent changes in brain plasticity. These studies should not be limited to the hippocampus and the hypothalamus but should also take into account other brain regions such as the basolateral amygdala (BLA) for its central role in the stress response and anxiety-like behavior.

## Concluding remarks and future directions

Our study indicates that the effects of PNS are clearly sex-dependent but do not lead to univocal results. While the experience of maternal stress during intrauterine life appears to promote metabolic dysfunction induced by a HFD at adulthood, the interaction between PNS and HFD is positive in male subjects only, and in agreement with the match-mismatch hypothesis, resulting in a reduction of anxious behaviors. Further studies are needed to detail the effects of *Bdnf* on the latter regulations taking into account the developmental stage, the brain region, specific *Bdnf* exons as well as epigenetic changes that create overall a highly complex picture (Boersma et al., 2014; Blaze et al., 2015; Braithwaite et al., 2015; Provencal and Binder, 2015; Cirulli, 2017).

There are some limitations in our study. First of all our conclusions about sex-dependent changes in emotional behavior in response to PNS and HFD are derived from only one behavioral test. Although the EPM is a very popular and reliable test to measure anxiety-like behavior, additional tests for anxiety and stress reactivity should be performed in future studies. Second, *Bdnf* levels were only measured in two brain areas one of which—the hypothalamus—is characterized by the presence of diverse neuronal populations with oftentimes opposing effects on metabolic outcomes. Thus, we cannot exclude that some of the observed changes as a result of PNS and HFD might be masked or neutralized when looking at the whole area. To strengthen the hypothesis that leptin resistance may predispose males toward an increased caloric intake, further markers should be examined in future studies such as SOCS-3 and PTP1B (Bjorbaek, 2009), in addition to those here assessed. Likewise, investigation of the expression level of NPY and POMC might help supporting the hypothesis of male subjects being more vulnerable to HFD**-**induced metabolic outcomes, including increased caloric intake.

## Author contributions

FC and MR designed the experiments, performed data interpretation and wrote this paper. PP performed the behavioral and metabolic assessments, performed statistical analysis, data interpretation and wrote this paper. AB supervised the experiments, performed statistical analysis and wrote the paper. CR performed the molecular assessment inherent to adiponectin, leptin and triglyceride measurements. VB and SC contributed to carry out metabolic assessments and tissue collection. AL and LL performed qRT-PCR to assess placental and brain gene expression.

### Conflict of interest statement

The authors declare that the research was conducted in the absence of any commercial or financial relationships that could be construed as a potential conflict of interest.
