# Supplementary material for: Long-Term Sex-Dependent Vulnerability to Metabolic challenges in Prenatally Stressed Rats
Source: Front Behav Neurosci. 2017 Jun 29;11:113. doi: 10.3389/fnbeh.2017.00113 (PMC5489562; doi:10.3389/fnbeh.2017.00113)
Supplement: Supplementary file 1 [file Image1.PDF]

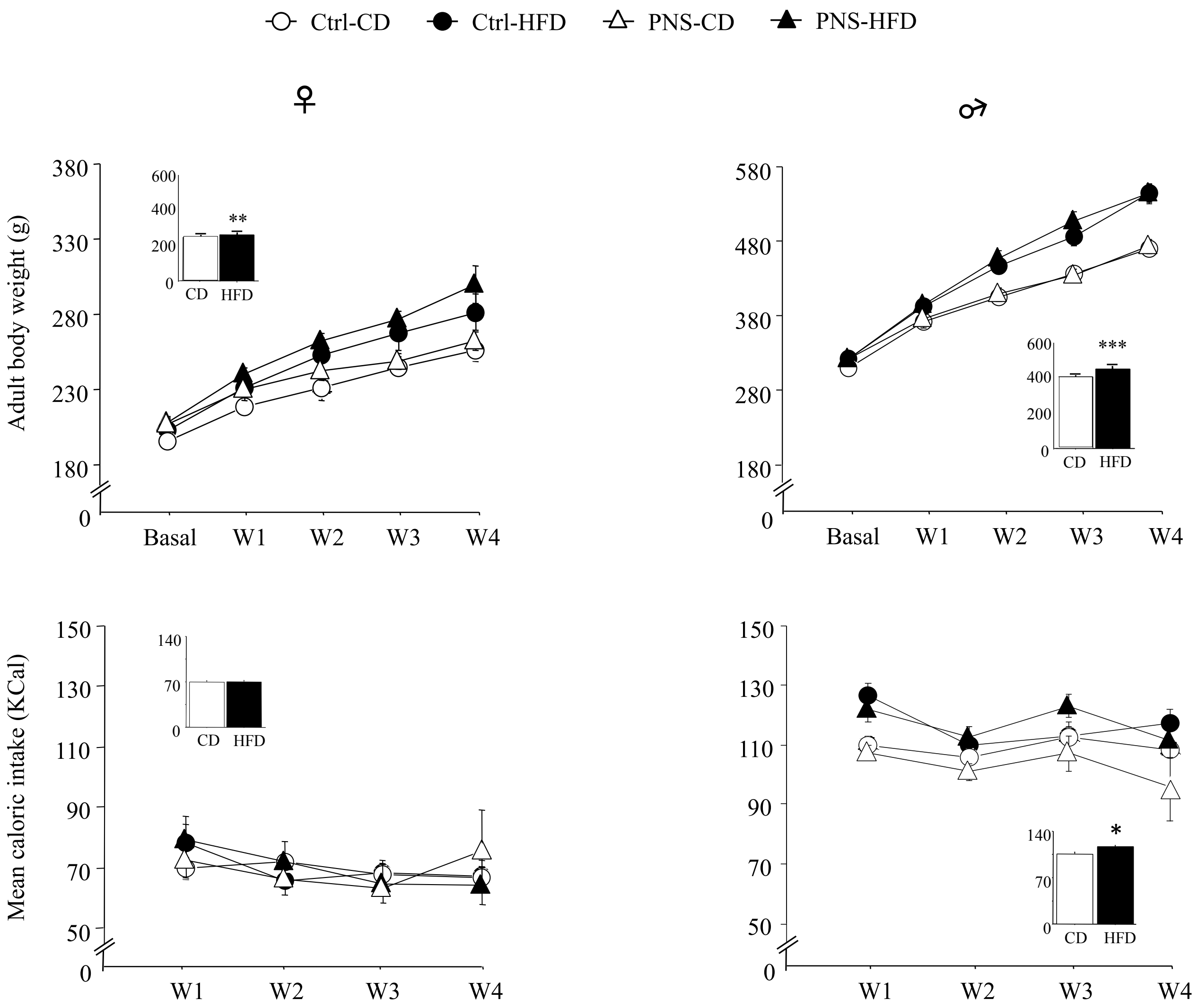

Supplementary Figure 1 Body weight and caloric intake in adult male and female subjects over the first four weeks of administration. All subjects fed HFD showed a significant increase in body weight. This effect is particularly apparent in male rats (upper panel). Male rats fed HFD are characterized by greater caloric intake (lower panel). * $p<0.05$; ** $p<0.01$; *** $p<0.0001$, main effect of Diet.
